# Aids to Nursing

**Published:** 1888-03-24

**Authors:** M. M. Basil

**Affiliations:** Author of "The Commoner Accidents to Life and Limb"


### Aids to Nursing.

By M. M. Basil, M.A., M.B., C.M., Author of "The Commoner Accidents to Life and Limb."

(*Continued from p.* 410.)

FOOD AND FEEDING.

The regular administration of food to the patient is one of the most important duties of the nurse in the sick room. In some cases of chronic ailment or of slight mental disease, it constitutes nearly all her duty. Some patients are more objects of care than subjects of cure, and the main point in their nursing is to feed them properly. Kitchen physic, judiciously and adroitly administered, is all they require. It was observable long ago that the use of whey and God's good providence constituted the essential treatment of typhus fever. The remark is applicable to many other diseases, and the nurse who will insist and persist until she gets the amount of food which has been ordered into her patient, is the one who will contribute to the largest number of recoveries. Mere stubborn insisting, however, will not do. Your zeal must here, as in all cases in the sick room, be tempered with knowledge.

It is better for the nurse to form no strong opinions as to which of her cases are hopeful and which utterly desperate. She should never relax her efforts to give medicine, food, and stimulants, no matter how hopeless all attempts may seem to her. Orders should be carried out as if life depended upon her alone. These remarks hold good for all the duties of a nurse, but they apply much more pointedly to the question of feeding. It should be kept in remembrance that when the doctor has done all he can, the life of the patient will frequently depend upon the nurse's patience and prudence in administering nourishment.

During meals there should be no hurry. All excitement in the ward must be avoided. "Unquiet meals make ill digestion," says an old proverb. No attempt should be made at "getting outside the dinner," as the Americans say. Patients who gulp down their food should be encouraged to chew. The appearance of haste on the part of the nurse will generally produce the same condition in the patients. Here, as in many things, like nurse like patient is the rule which will be established, whether you like it or not.

Plates, forks, bread and food should be presented to the patients in a quiet, civil way, not in an off-hand manner. Heavy plates must not be trusted into the hands of the patient. A proper table which can be used in bed is a great boon to many. A glass rod, bent nearly at right angles, is an efficient means for enabling a patient, when lying in the recumbent position, to drink fluids off a tumbler. It is a much more convenient arrangement than an ordinary feeding cup.

If any patient is helpless or of dirty habits, he should be kept separate from the others in a ward. Keep the patient dry under the chin, and never use the feeding cup without first arranging some cloth to catch any drops that may be spilt. The bottom of the cup also should be dry. To secure this object, please remember never to fill any cup, jug, ewer, or medicine bottle quite to the top. It is difficult to pour off any fluid from such a vessel without spilling it; and you must always make allowance for accidental jerks from shakiness, coughing, and the like.

Food should be out of sight and out of smell during its preparation. It should be brought to the patient at the right time and in right quantity, rather less than more, and whether eaten or not, it should be removed out of the sick room at once. The mere sight of food left in the room is nauseating to many patients, especially to such as are suffering from acute diseases. Even liquid food, such as milk, beef-tea, soups, etc., should not be left by the sick-bed. Most of these nutrients are admirable feeding-grounds for disease germs, and, at the temperature of the sick room, they act as delicately adapted incubators for their cultivation. So that the nutrient becomes very soon a dangerous magazine of infection. The little leaven leavens the whole lump in a very alarming manner. After remaining in the sick room for a time it is almost sure to prove injurious if given to a patient susceptible to the poison. An additional dose of the poison is not likely to be anything but harmful to the patient who is already suffering from its effects.

These considerations will make it clear to you that, unless you are extremely cautious, you may unwittingly either poison a patient through his food, or add to the risks of a disease which is itself the result of previous poisoning. Perhaps this lesson may be remembered by you more easily if I mention that a whole series of ailments are now known as *milk diseases*, because milk, the most commonly used of these nutrient media, has been found to be a frequent means of spreading them as epidemics.

For reasons which will be quite evident to you now, you should never eat in the same room with the patient. You should never cool anything by blowing on it. Besides the fact that it may be disagreeable to your patient, there is risk of communicating disease to him, should it happen that you are in the early stages of one of the infectious fevers. Another general rule is never to taste food before giving it to the patient. In exceptional conditions, however, as when a suspicious patient takes ideas of secret poisoning into his head, it is not only allowable, but beneficial to taste his food before offering it to him.

As a rule you should note down beforehand the hours when medicine and food fall due, and attempt to carry out your programme. In many diseases, however, you must modify these arrangements considerably. If the patient will not take food at the usual time, watch and find out any hour when he could take it by preference and then present it to him quickly. In serious disease you must not bother a patient by asking him whether he would like this and that. Do the thinking and the making up of mind for him. Prepare what you know is suitable for his case and present it to him.

Sometimes a patient will refuse food from one nurse and take it from another. An attendant of the opposite sex will often succeed in feeding a patient when others have given up the attempt. All such freaks should be observed and turned to the advantage of the patient. I need hardly say that it would be very unkind to take any offence at these vagaries of a sick man.

You cannot be too careful of the quality of the sick diet. Its appearance, smell, and taste must all receive minute attention. Never present what is repugnant to sight. It is far preferable to allow the patient to miss the appointed meal entirely. In the tea there should be no leaves or other articles floating about, no sand should be found in the porridge. You should never attempt to remove foreign bodies, especially flies, out of the food in presence of the patient. Vanish out of his sight; the quicker the better. The confidence gained by such minute attention to details will be of service in the future nursing of the case.

Prostrate people must be fed tenderly. Meat, bread, potatoes, etc., must be cut up for them. The strength of will or power of resolution, among other things, suffers in all serious or prolonged diseases, and thus it often happens that the mere inability to screw up courage to the chopping and chewing point deters a patient from taking sufficient food.

Little and often is the rule in feeding many patients, but it must not be too little nor too often. The patient should not be disturbed too frequently, and the stomach should be allowed to get rest so as to recruit its power. There is an impression abroad that the tinier the morsel the easier it is to swallow. But the fact is that very tiny morsels, like tiny pills, cannot be easily got over, because the muscles of swallowing can get no purchase or grasp of them. Besides, ridiculously small morsels harass a patient and tire out the muscles of mastication uselessly.

*(To be continued.)*

